# Decoupling of Respiratory Virus Positivity and Host Inflammatory Response: A 16-Year Longitudinal Study

**DOI:** 10.3390/microorganisms14040908

**Published:** 2026-04-17

**Authors:** Sung Hun Jang, Jeong Su Han, Jae Kyung Kim

**Affiliations:** 1 Department of Medical Laser, Graduate School of Medicine, Dankook University, Cheonan-si 31116, Republic of Korea; well8143@naver.com; 2 Department of Biomedical Laboratory Science, College of Health Sciences, Dankook University, Cheonan-si 31116, Republic of Korea; jshan1162@naver.com

**Keywords:** C-reactive protein, inflammation, respiratory tract infections, pandemics, polymerase chain reaction

## Abstract

Given limited evidence on temporal changes in pathogen detection patterns and hospital-based inflammatory burden across the pandemic transition, this study examined their long-term relationship using respiratory multiplex PCR positivity and concurrent C-reactive protein (CRP) levels. We analyzed 19,002 episodes linking respiratory multiplex PCR (mPCR) results and concurrent CRP from October 2008 to December 2024. Pre-pandemic, pandemic, and post-pandemic changes in monthly testing volume, positivity rate, median CRP, high and extreme inflammation by mPCR status, and the correlation between positivity rate and median CRP were assessed. mPCR positivity decreased from 60.62% (pre-pandemic) to 22.45% (pandemic) and remained low at 25.95% thereafter, whereas the median CRP increased from 0.94 to 3.35 and 5.97 mg/dL, respectively. After January 2020, testing volume and positivity rate decreased, whereas the median CRP increased. High inflammation increased from 13.78% to 27.93% and 38.98% in mPCR-negative episodes, and from 4.61% to 7.20% and 27.66% in mPCR-positive episodes, remaining consistently lower in the latter. Monthly positivity rate was strongly negatively correlated with median CRP. Overall, respiratory virus positivity declined, whereas CRP-based inflammatory burden increased, indicating divergent temporal trends across the pandemic transition. These findings should be interpreted descriptively, not causally, as reflecting divergent temporal trajectories of pathogen detection and inflammatory burden.

## 1. Introduction

Respiratory infections are among the most common diagnostic challenges in outpatient, emergency, and inpatient care settings. At the time of initial presentation, clinicians must make decisions under substantial uncertainty, and simultaneously consider the natural course of viral illness, possibility of bacterial infection, and potential harms associated with unnecessary antibiotic use [1]. C-reactive protein (CRP), a biomarker of the host inflammatory response, has been widely used as an adjunctive marker to rapidly assess inflammatory burden and support decisions regarding antibiotic prescriptions for patients with respiratory symptoms [2]. Point-of-care testing for CRP reduces antibiotic prescriptions for non-severe acute respiratory infections in primary care settings, consistent with systematic reviews and meta-analyses [3].

Advances in molecular diagnostics have led to the rapid clinical adoption of respiratory multiplex PCR (mPCR) panels [4]. By simultaneously detecting a wide range of respiratory pathogens from a single specimen, mPCR has improved both speed and comprehensiveness of diagnoses, and systematic meta-analyses have confirmed the high diagnostic accuracy of major commercially available systems [5]. Early pathogen identification using mPCR may influence antibiotic use, infection control practices, and clinical operational metrics [6]. Studies evaluating the impact of respiratory panel testing on antibiotic use patterns in children with acute febrile respiratory illness further suggest that test results can be meaningfully integrated into real-world clinical decision-making [7].

However, pathogen detection data cannot be assumed to correspond directly to host inflammatory burden. CRP does not distinguish between viral and bacterial infection; even in patients infected with the same pathogen, the magnitude and pattern of the inflammatory response may vary according to underlying comorbidities, site of infection, complications, coinfection, timing of presentation, and therapeutic intervention [8]. Moreover, mPCR results are influenced by the panel composition and selection of the tested population; therefore, an mPCR-negative result does not necessarily indicate the absence of viral infection. Rather, it may reflect pathogens not included in the panel, suboptimal timing of specimen collection, limited upper-respiratory detection in lower respiratory tract infection, noninfectious inflammation, or concomitant bacterial infection [9]. Accordingly, examining mPCR results (pathogen detection) and CRP levels (hospital-based inflammatory burden) on the same long-term time axis may help characterize how these two distinct clinical signals changed relative to one another within the same testing environment.

This issue became even more pronounced during the COVID-19 pandemic. Through non-pharmaceutical interventions (NPIs), changes in contact structure, and reorganization of healthcare utilization, the pandemic simultaneously altered both the respiratory virus ecosystem and the diagnostic environment. Surveillance studies have reported unprecedented reductions in the detection of multiple respiratory viruses, including influenza and respiratory syncytial virus (RSV), around 2020 [10,11]. However, this phenomenon likely represented more than a simple decline in circulation; in conjunction with changes in testing volume, patient selection, testing pathways (outpatient, emergency, and inpatient), infection-control policies, and treatment thresholds, it may also have altered the clinical meaning of a given mPCR result. Furthermore, during the early phase of the pandemic, there was a period in which conventional respiratory panels did not include SARS-CoV-2, and later, respiratory panels incorporating SARS-CoV-2 targets were introduced under Emergency Use Authorization. This transition raises the possibility that the diagnostic implications of an mPCR-negative result, including the possibility of pathogens not covered by the panel, separation of testing pathways, and shifts in the presenting patient population, may have changed over time [12]. Review articles summarizing the impact of the pandemic on respiratory virus epidemiology and diagnostic testing patterns have similarly discussed the possibility that pandemic-associated changes in testing and circulation structures may persist over the long term [13].

The clinical significance of CRP was re-emphasized during the pandemic. In COVID-19, CRP was repeatedly reported to be associated with disease severity and prognosis [14]. Additionally, the concept of immune debt or an immunity gap was proposed, suggesting that reduced exposure during the pandemic may have led to an accumulation of susceptibility and, consequently, altered patterns of subsequent viral circulation. This has prompted continued discussion regarding the re-emergence of seasonal viruses and changes in clinical burden during the post-pandemic period (2023–2024) [15]. Such epidemiologic transitions may reshape the detection–inflammation relationship independently of simple changes in viral circulation, by altering healthcare utilization, testing selection, and the clinical spectrum of presenting patients.

Previous studies have often focused on short-term comparisons centered on specific viruses or age groups, or been limited to descriptive changes in detection rates. Quantitative evidence integrating pathogen detection and host inflammatory response over the same long-term time axis remains limited. In a setting where testing volume, patient selection, testing pathways, and the contextual use of diagnostic panels may all have changed across the pre-pandemic, pandemic, and post-pandemic periods, mPCR positivity alone may not adequately estimate the burden of high inflammation. As testing pathways, panel coverage, and the composition of the tested population changed across the pre-pandemic, pandemic, and post-pandemic periods, it is important to determine whether pathogen detection patterns and hospital-based inflammatory burden continued to show similar temporal behavior over time. More specifically, it is important to determine how CRP-based inflammatory burden changed as pathogen detection rates declined or were reconfigured, and whether these temporal changes differed between mPCR-positive and mPCR-negative episodes.

Accordingly, this study linked respiratory mPCR episodes and concurrently measured CRP values from a single tertiary-care hospital between October 2008 and December 2024 to examine the long-term temporal relationship between pathogen detection patterns and hospital-based inflammatory burden across the pre-pandemic, pandemic, and post-pandemic periods. Respiratory mPCR was used to represent pathogen detection within the testing panel in place at the time, whereas CRP was used to reflect the accompanying inflammatory burden measured in the same hospital-based setting. Using a time-series approach, we examined whether these two signals changed in parallel or followed different temporal trajectories over time, with a focus on their longitudinal temporal relationship in the hospital-based setting.

## 2. Materials and Methods

### 2.1. Study Design

This study was a retrospective observational study and long-term time-series analysis that linked the results of a 15-target respiratory mPCR panel with serum CRP values measured within the same clinical episode at Dankook University Hospital, a tertiary-care hospital in Cheonan, Republic of Korea, from October 2008 to December 2024. By integrating these data, we quantified annual and monthly changes in virus-specific detection rates and CRP-based indicators. The study design and reporting followed the Strengthening the Reporting of Observational Studies in Epidemiology guidelines.

### 2.2. Study Population and Inclusion Criteria

The primary unit of analysis was defined as a testing episode corresponding to a single specimen collection. Because the aim of this study was to describe episode-level testing patterns and linked inflammatory burden in a real-world hospital setting over time, repeated testing episodes from the same patient during the study period were included in the primary analysis as episode-based observations. To evaluate the potential influence of within-patient repetition, additional sensitivity analyses were performed using both a patient-level first-episode dataset and generalized estimating equation (GEE) models with a unique patient identifier as the clustering variable. Each episode was treated as a specimen-based observation and individually analyzed in annual and monthly aggregation as well as in time-series analyses. Among all mPCR tests performed during the study period (October 2008 to December 2024), episodes were included if a corresponding CRP result linked to the same testing episode was available in the laboratory information system (LIS). The key episode-level variables collected were testing time point (including result date for annual and monthly aggregation), positive or negative status for each pathogen target included in the panel, number of detected pathogens (single detection vs. codetection), and CRP concentration (mg/dL). The analysis focused on how temporal structure (year/month) and combinations of pathogen detection were reflected in host inflammatory markers.

Respiratory viruses included in the analytical framework of this study were influenza A (Flu A), influenza B (Flu B), respiratory syncytial virus (RSV) A, RSV B, human metapneumovirus (hMPV), parainfluenza virus (PIV)-1, PIV-2, PIV-3, human rhinovirus (HRV), human coronavirus (HCoV)-229E, HCoV-OC43, HCoV-NL63, adenovirus (ADV), enterovirus (ETV), and human bocavirus (BOCA). Not all targets were available throughout the entire study period; HCoV-NL63 and BOCA were included from 2015 to 2023, and ETV from 2018 to 2023. Accordingly, virus-specific analyses were performed only during the periods in which each target was included in the clinical mPCR panel.

### 2.3. Respiratory Virus Detection

Specimens were tested immediately after collection whenever possible. If immediate testing was not feasible, specimens were stored at 4 °C and analyzed within 24 h. Viral RNA was extracted using the QIAamp Viral RNA Mini Kit (QIAGEN, Hilden, Germany). From 2008 to 2012, respiratory viruses were detected using the Seeplex RV series mPCR assay (Seegene, Seoul, Republic of Korea), which was interpreted using gel electrophoresis following conventional PCR. From 2013 onward, the AdvanSure RV and RV-Plus real-time RT-PCR kits (LG Chem, Seoul, Republic of Korea) were used, and amplification was performed on the SLAN Real-Time PCR System (LG Chem). All assays were performed in accordance with the manufacturers’ standard protocols and internal quality-control procedures.

### 2.4. Measurement of Serum CRP

All CRP measurements were performed using an automated immunoturbidimetric assay on the Cobas 8000 modular analyzer (Roche Diagnostics, Mannheim, Germany) with the dedicated reagent Tina-quant C-Reactive Protein Gen.3 (Roche Diagnostics). Although the manufacturer reports the analytical range in mg/L, CRP results are reported in mg/dL in the laboratory information system (LIS) of our institution; therefore, all CRP values were standardized and analyzed in mg/dL in this study. Accordingly, the reportable range in our laboratory was 0.03–70 mg/dL, with a direct measurement range of 0.03–35 mg/dL without dilution. When the measured value exceeded 35 mg/dL, the analyzer automatically performed a 1:2 dilution and remeasurement, extending the upper reportable limit to 70 mg/dL. In this study, the final reported values stored in the LIS were directly used for analysis, without any additional adjustment or substitution, such as replacement with half the limit of quantification. For ease of interpretation, mg/dL values may be converted to mg/L by multiplying by 10 when needed. After clotting, blood samples were immediately centrifuged to separate serum, stored at 2–8 °C, and analyzed within 24 h. Internal and external quality-control procedures were maintained throughout the study period to ensure analytical consistency across years.

### 2.5. Statistical Analyses

After monthly time-series summary data were constructed, interrupted time-series analysis using linear segmented regression was performed with breakpoints at January 2020 and January 2023 to estimate changes in level and slope. Newey–West heteroscedasticity- and autocorrelation-consistent standard errors with a lag of 1 were used to account for autocorrelation and heteroscedasticity in the monthly data. High inflammation was defined as CRP ≥ 10 mg/dL. Extreme inflammation was defined as CRP values at or above the 99.5th percentile (P99.5) of the CRP distribution among mPCR-negative episodes from 2008 to 2024. Period-wise differences in the proportions of high and extreme inflammation were evaluated separately for mPCR-negative and mPCR-positive episodes using Pearson’s chi-square test, and linear trends across the ordered periods were assessed using the Cochran–Armitage trend test. As a sensitivity analysis for repeated observations from the same patient, GEE models with a binomial distribution, logit link, and exchangeable correlation structure were additionally fitted for the binary outcomes of CRP ≥ 10 mg/dL and CRP ≥ P99.5, using a unique patient identifier as the clustering variable. In addition, the principal descriptive comparisons were repeated after restricting the dataset to the first eligible episode per patient during the study period. Between-period differences in virus-specific detection were assessed using the chi-square test of independence (omnibus test), with multiple comparisons adjusted by the Benjamini–Hochberg false discovery rate method. In the monthly summary data, concordance or decoupling between mPCR positivity rates and CRP-based indicators was quantified using Spearman’s correlation analysis. All tests were two-sided, and *p* < 0.05 was considered statistically significant. All analyses were performed using R software (Version 4.3.3; R Foundation for Statistical Computing, Vienna, Austria).

### 2.6. Ethical Considerations

This retrospective study used anonymized data and was conducted in accordance with the ethical principles of the Declaration of Helsinki of 1975, as revised in 2013. The study was approved by the Institutional Review Board of Dankook University Hospital (approval no. DKUH 2026-01-013-001; approval date: 5 March 2026), and the requirement for informed consent was waived because of the retrospective design and use of de-identified data.

## 3. Results

### 3.1. Overview of the Study Population and Temporal Changes in Testing Structure Across Study Periods

From October 2008 to December 2024, 19,002 linked respiratory virus PCR–CRP testing episodes were identified at Dankook University Hospital over a 16-year and 3-month period. During the same study period, a total of 23,284 respiratory mPCR episodes were identified, of which 19,002 (81.61%) had linked CRP results and were included in the final analysis, whereas 4282 episodes (18.39%) were excluded because no corresponding CRP result was available. When the study period was divided into the pre-pandemic (2008–2019), pandemic (2020–2022), and post-pandemic (2023–2024) phases, changes in viral detection patterns and codetection structure were observed after the transition in 2020 (Table 1).

Episodes with detection of at least one respiratory virus decreased from 60.62% (9438/15,569) in the pre-pandemic period to 22.45% (486/2165) during the pandemic period and remained low at 25.95% (329/1268) in the post-pandemic period. Codetection of two or more viruses also declined, from 14.21% (2212/15,569) in the pre-pandemic period to 6.65% (144/2165) during the pandemic period, and remained at a similar level thereafter (6.39% [81/1268]) in the post-pandemic period.

With respect to the distribution of the number of detected viruses, single-virus detection (7226 episodes) and two-virus detection (1884 episodes) were relatively common in the pre-pandemic period. In contrast, during and after the pandemic period, the proportion of negative episodes increased (1679 during the pandemic period and 939 in the post-pandemic period), whereas the frequencies of single, double, and higher-order detections decreased overall.

However, multiple-virus detection did not disappear completely. Triple-virus detection was observed in 300, 24, and 14 episodes in the pre-pandemic, pandemic, and post-pandemic periods, respectively. Four-virus detection occurred in 28, 4, and 3 episodes in the pre-pandemic, pandemic, and post-pandemic periods, respectively, and one episode with six-virus detection was identified in the post-pandemic period.

**Figure 1:**
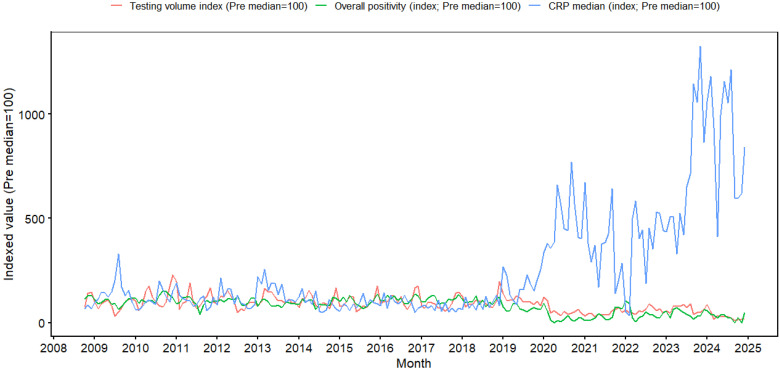
Long-term monthly trends in indexed respiratory multiplex PCR testing volume, overall positivity, and median CRP, 2008–2024. Monthly testing volume, overall positivity, and median CRP were indexed to the pre-pandemic monthly median (October 2008 to December 2019 = 100) using the formula: index = (monthly value/pre-pandemic median) × 100. Overall positivity is presented as an indexed value rather than as a raw percentage. CRP, C-reactive protein.

The median CRP level across all specimens increased stepwise from 0.94 mg/dL in the pre-pandemic period to 3.35 mg/dL during the pandemic period and 5.97 mg/dL in the post-pandemic period (Figure 1).

An immediate shift in testing structure was observed after the pandemic transition point in January 2020. The monthly testing volume (level change, −51.34 episodes/month; 95% CI, −70.11 to −32.57; *p* < 0.001) and monthly positivity rate (level change, −31.86 percentage points; 95% CI, −42.89 to −20.83; *p* < 0.001) significantly decreased in January 2020. In contrast, the monthly median CRP level significantly increased at the same time point (level change, +2.61 mg/dL; 95% CI, 1.99 to 3.24; *p* < 0.001). In the additional transition in January 2023, no significant immediate level changes were observed in testing volume or positivity rate, whereas the median CRP level showed a further increase (level change, +3.51 mg/dL; 95% CI, 2.04 to 4.97; *p* < 0.001) (Table 2).

### 3.2. Stepwise Increase in CRP Levels and the Predominant Contribution of the mPCR-Negative Group

Across the pre-pandemic, pandemic, and post-pandemic periods, the median CRP level in the respiratory virus-negative group increased stepwise from 0.94 mg/dL during the pre-pandemic period to 3.35 mg/dL during the pandemic period and 5.95 mg/dL in the post-pandemic period. During the same periods, the median CRP levels in the single-positive and multiple-positive groups also increased; however, within each period, the three groups showed broadly comparable median values, indicating that the central tendency of the distribution did not substantially differ across groups.

In contrast, mean CRP levels showed a more pronounced elevation in the negative group. In the pre-pandemic period, the mean CRP level in the negative group was 4.21 mg/dL (95% CI, 4.05–4.36), which was higher than that in the single-positive (2.29 mg/dL; 95% CI, 2.19–2.39) and multiple-positive (2.08 mg/dL; 95% CI, 1.94–2.23) groups, corresponding to absolute differences of +1.92 mg/dL (1.84-fold) and +2.13 mg/dL (2.02-fold), respectively. This pattern was maintained and became more marked during the pandemic period, when the mean CRP level in the negative group reached 7.38 mg/dL (95% CI, 7.01–7.76), exceeding that of the single-positive group (3.09 mg/dL; 95% CI, 2.46–3.71) by +4.29 mg/dL (2.39-fold) and that of the multiple-positive group (1.47 mg/dL; 95% CI, 1.05–1.89) by +5.91 mg/dL (5.02-fold). In the post-pandemic period, the negative group continued to show the highest mean CRP level at 9.45 mg/dL (95% CI, 8.90–10.00), remaining higher than the single-positive group (7.56 mg/dL; 95% CI, 6.56–8.57) by +1.89 mg/dL (1.25-fold) and multiple-positive group (4.75 mg/dL; 95% CI, 3.26–6.25) by +4.70 mg/dL (1.99-fold).

**Figure 2:**
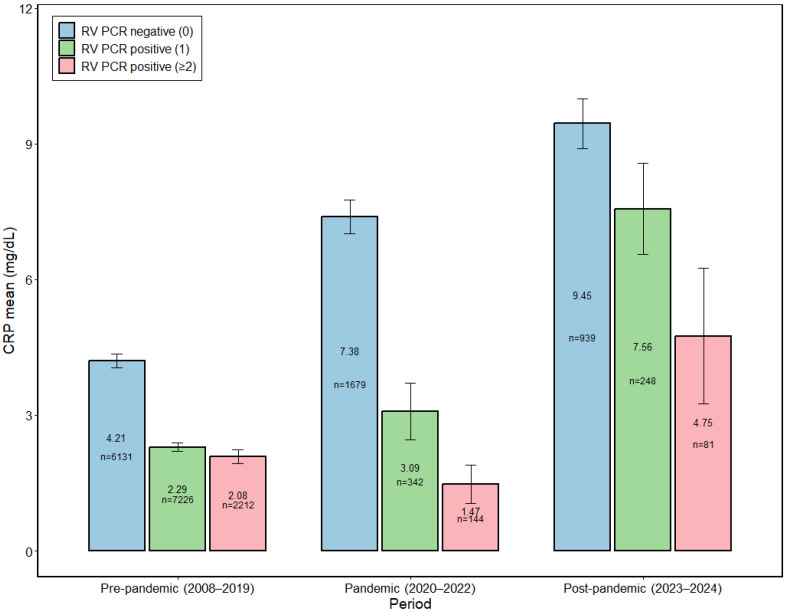
Period-specific mean C-reactive protein (CRP) levels using multiplex respiratory virus (RV) PCR detection status (2008–2024). Bars show mean CRP concentrations (mg/dL) for each period, pre-pandemic (2008–2019), pandemic (2020–2022), and post-pandemic (2023–2024), stratified by multiplex mPCR result: negative (0), positive (1), and positive (≥2). Values above bars indicate the mean CRP and “*n*” inside bars denotes the number of episodes. Multiplex results are grouped as 0/1/≥2 detections; “≥2” includes co-detection of two or more viruses per episode (two to six viruses as detailed in Table 1).

Overall, across all three periods, the highest mean CRP levels were consistently observed in the virus-negative group. This pattern suggests that, alongside the overall upward shift in mean CRP levels toward the post-pandemic period, the contribution of the negative group became increasingly prominent (Figure 2). To provide descriptive context for the grouped categories shown in Figure 2, the distribution of respiratory virus detections within the 1-positive and ≥2-positive groups is summarized in Table S1. HRV was the most frequent virus in both groups; in the ≥2-positive group, ADV was also prominently represented.

### 3.3. High-Inflammation Burden in mPCR-Negative Episodes, with Comparison to mPCR-Positive Episodes

**Figure 3:**
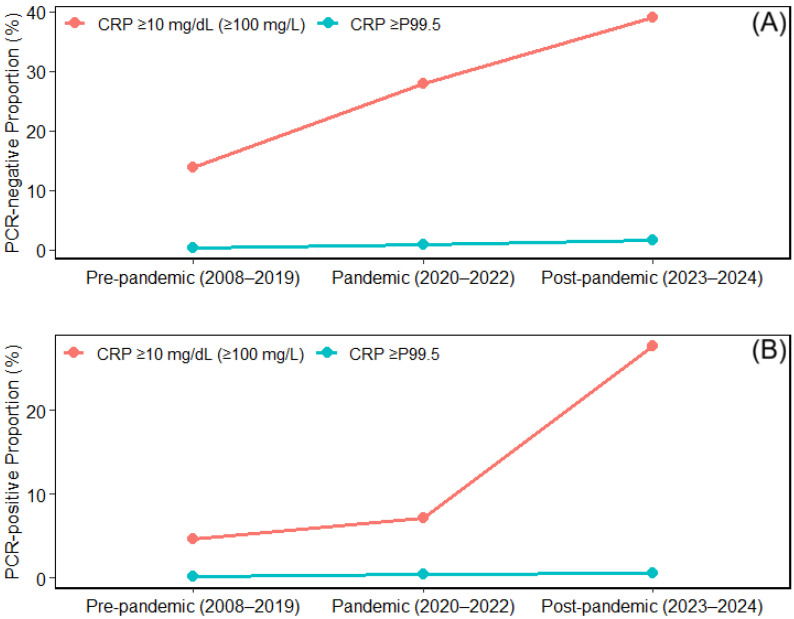
Period-specific proportions of CRP ≥ 10 mg/dL and CRP ≥ P99.5 in respiratory virus PCR-negative and PCR-positive episodes. (**A**) PCR-negative episodes; (**B**) PCR-positive episodes. Period-specific proportions (%) of episodes with CRP ≥ 10 mg/dL and CRP ≥ P99.5 are shown for the pre-pandemic (2008–2019), pandemic (2020–2022), and post-pandemic (2023–2024) periods.

During the study period, the proportion of high-inflammatory cases, defined as CRP ≥ 10 mg/dL, increased significantly across the pre-pandemic, pandemic, and post-pandemic periods in both respiratory mPCR-negative and mPCR-positive episodes (Table 3; Figure 3). Among mPCR-negative episodes, the proportion increased from 13.78% (845/6131) in the pre-pandemic period to 27.93% (469/1679) during the pandemic period and further to 38.98% (366/939) in the post-pandemic period (chi-square *p* < 0.001; trend *p* < 0.001). Among mPCR-positive episodes, the corresponding proportion increased from 4.61% (435/9438) to 7.20% (35/486) and 27.66% (91/329), respectively (chi-square *p* < 0.001; trend *p* < 0.001).

For extreme inflammation, defined as CRP ≥P99.5, mPCR-negative episodes showed an increase from 0.28% (17/6131) in the pre-pandemic period to 0.77% (13/1679) during the pandemic period and 1.49% (14/939) in the post-pandemic period (chi-square *p* < 0.001; trend *p* < 0.001). In mPCR-positive episodes, the corresponding proportions were 0.16% (15/9438), 0.41% (2/486), and 0.61% (2/329), respectively. Although the overall between-period difference for CRP ≥ P99.5 in mPCR-positive episodes did not reach statistical significance (chi-square *p* = 0.0873), a significant increasing trend across the ordered periods was observed (trend *p* = 0.0275).

To assess whether repeated testing episodes from the same patient influenced these findings, we additionally performed GEE analyses accounting for within-patient clustering (Table S2). The overall direction of the findings remained unchanged. For CRP ≥10 mg/dL, compared with the pre-pandemic period, the odds were significantly higher during the pandemic period (OR, 2.43; 95% CI, 2.13–2.76) and post-pandemic period (OR, 4.01; 95% CI, 3.45–4.66) in PCR-negative episodes, and were also higher during the pandemic period (OR, 1.61; 95% CI, 1.13–2.30) and post-pandemic period (OR, 7.30; 95% CI, 5.59–9.53) in PCR-positive episodes. In an additional sensitivity analysis restricted to the first eligible episode per patient, the same overall directional pattern was observed (Table S3).

### 3.4. Declining Virus-Specific Detection Rates and Their Temporal Relationship with Inflammatory Markers

**Figure 4:**
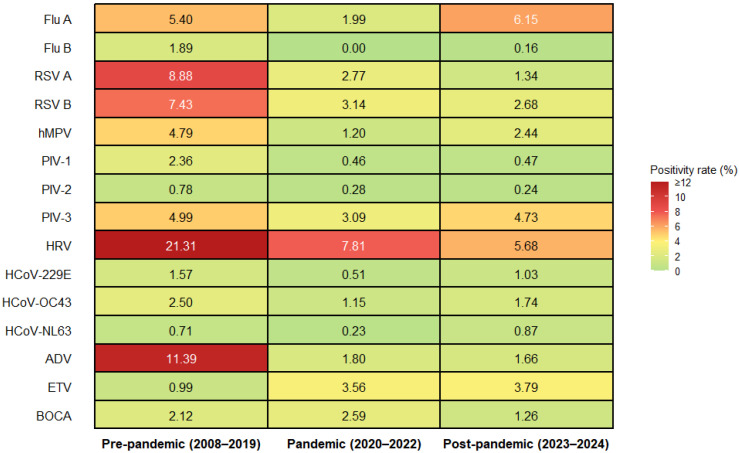
Heatmap of period-specific positivity rates for 15 respiratory viruses across the pre-pandemic, pandemic, and post-pandemic periods. Each cell displays the period-specific positivity rate (%), and color intensity represents the magnitude of positivity (green to red, low to high). For targets added later to the multiplex PCR panel (HCoV-NL63/BOCA: 2015–2023; ETV: 2018–2023), positivity rates were calculated only for the corresponding panel-covered periods (i.e., periods before panel inclusion were excluded from the denominator). Abbreviations: Flu A, influenza A; Flu B, influenza B; RSV A/B, respiratory syncytial virus A/B; hMPV, human metapneumovirus; PIV-1/2/3, parainfluenza virus type 1/2/3; HRV, human rhinovirus; HCoV-229E/OC43/NL63, human coronavirus 229E/OC43/NL63; ADV, adenovirus; ETV, enterovirus; BOCA, human bocavirus.

Comparison of positivity rates across the pre-pandemic, pandemic, and post-pandemic periods showed significant between-period differences for 15 viruses. Significant changes were observed for human rhinovirus (HRV), adenovirus (ADV), RSV A, ETV, RSV B, human metapneumovirus (hMPV), influenza B, parainfluenza virus (PIV)-1, influenza A, PIV-3, HCoV-OC43, and HCoV-229E (all *p* < 0.001), as well as for PIV-2 (*p* = 0.005), HCoV-NL63 (*p* = 0.027), and BOCA (*p* = 0.034). Among these, HRV declined from 21.31% to 5.68%, and ADV from 11.39% to 1.66%, indicating particularly marked reductions (Figure 4). Significant decreases across periods were also observed for influenza B, RSV A/B, and PIV-2, and overall, the dominant pattern was one of decline or persistence at a low level. ETV, hMPV, PIV-1, HCoV-NL63, HCoV-OC43, HCoV-229E, and BOCA remained at low levels even after their decline during the pandemic period.

However, the direction of change was not uniform across all viruses. Influenza A decreased during the pandemic period but rebounded to 6.15% in the post-pandemic period, exceeding its pre-pandemic level. Similarly, PIV-3 increased to 4.73% in the post-pandemic period. Overall, most viruses showed lower detection rates in the post-pandemic period than in the pre-pandemic period.

In contrast to the declining trends observed for most virus-specific positivity rates, the median CRP level increased stepwise over time. The overall median CRP level increased from 0.94 mg/dL during the pre-pandemic period to 3.35 mg/dL during the pandemic period and further to 5.97 mg/dL in the post-pandemic period (Table 1). Therefore, CRP continued to increase while virus detection rates overall declined or were redistributed over time.

### 3.5. Monthly Time-Series Correlation Analysis: Negative Correlation Between Positivity Rate and Median CRP

Comparison of the monthly overall respiratory virus positivity rate (%) and median CRP level (mg/dL) from October 2008 to December 2024 showed that the two indicators generally moved in opposite directions throughout the study period. During intervals in which the monthly positivity rate increased, the median CRP level tended to remain relatively low, whereas during intervals in which the positivity rate declined, the median CRP level was repeatedly observed to remain relatively high. This inverse temporal pattern was consistently evident even when the entire 195-month time series was analyzed as an integrated dataset, with a strong negative association between the two indicators in the overall analysis (ρ = −0.744, *p* < 0.001).

After 2020, during the pandemic period, the monthly positivity rate showed a marked decline compared with the seasonal peaks observed in earlier years, whereas the median CRP level frequently remained elevated or increased further (Table S4). From a long-term time-series perspective, these findings indicate a lack of parallelism between the positivity rate and median CRP level, with an overall inverse temporal relationship.

## 4. Discussion

This study, based on 16 years of long-term time-series data from a single tertiary-care hospital, shows that the relationship between respiratory virus detection and host inflammatory burden did not remain constant over time across the transitions spanning the pre-pandemic, pandemic, and post-pandemic periods. The central finding was that, although mPCR-based respiratory virus detection rates were restructured to an overall lower level after the pandemic period, the inflammatory burden represented by CRP shifted in the opposite direction, showing progressive elevation. These findings suggest that pathogen detection and host-response information may not always function as interchangeable measures along a single axis, but may instead reflect distinct dimensions that evolved over time in the post-pandemic setting. This pattern was consistently observed in the interrupted time-series analysis. At the January 2023 transition point, no significant immediate level changes were observed in testing volume or positivity rate, whereas the median CRP level significantly increased at both January 2020 and January 2023 breakpoints. The marked reduction in the circulation of multiple respiratory viruses during the pandemic period, likely driven by NPIs and changes in contact patterns, is consistent with previous studies from Korea, the United Kingdom, and the United States [16,17,18]. Additionally, our data suggest that the low detection level established during the pandemic period may have been sustained, at least in part, into the post-pandemic period. Furthermore, the stepwise increase in CRP suggests that changes in inflammatory burden may not be fully accounted for solely by the simple recovery or decline of pathogen detection volume.

In summary, the main findings differed clearly across periods: the pre-pandemic phase was characterized by higher respiratory virus positivity and lower CRP, whereas the pandemic and post-pandemic phases showed persistently reduced positivity together with progressively increased CRP.

However, because SARS-CoV-2 was not included in the respiratory mPCR panel during the early phase of the pandemic and SARS-CoV-2 results were not available as a uniformly linked variable across the full study dataset, the exact proportion of COVID-19-positive cases within the cohort, including the mPCR-negative subgroup, could not be determined. Accordingly, some undetected or separately tested SARS-CoV-2 infections may have been included in the mPCR-negative group during that interval and may have contributed to the elevated CRP levels observed during the pandemic period [12]. Therefore, mPCR negativity should be interpreted not as the absence of respiratory viral infection, but rather as a residual category that may include infections not captured by the diagnostic panel available at the time. Collectively, changes in detection rates before and after the pandemic reflect changes in epidemic magnitude, as well as the combined effects of NPI-driven transmission suppression, changes in panel targets, restructuring of testing requests, and shifts in the composition of the tested population [19].

The stepwise increase in CRP and the predominant contribution of the mPCR-negative group become even clearer when considered from the perspective of distributional shape. The finding that median CRP levels did not substantially differ between negative and positive groups within the same period suggests that differences in the center of the distribution may have been limited. In contrast, the consistently higher mean CRP observed in the negative group implies a heavier right tail and a greater relative frequency of high-value observations in that group. This pattern is also compatible with the characteristics of a tertiary-care hospital dataset enriched for patients with greater clinical complexity and disease severity [20]. Even when no virus is detected by mPCR, CRP is often repeatedly measured in actual clinical practice for severity assessment, differential diagnosis, and monitoring of treatment response or recovery [21,22]. This group may therefore include a relatively higher proportion of severely ill patients or patients with complex comorbid conditions. Thus, the elevated mean CRP in the mPCR-negative group is better interpreted as a hospital-based pattern potentially shaped by selection bias, case-mix restructuring, panel non-coverage, and other unmeasured infectious or noninfectious inflammatory conditions, rather than as evidence of any single unmeasured pathogen.

Accordingly, the mPCR-negative/high-CRP group should be interpreted as a clinically heterogeneous category rather than a uniform residual disease group, and caution is warranted when inferring its underlying cause.

This interpretation is also consistent with the changing distribution of high inflammatory burden within the negative group. Previous studies have similarly defined CRP ≥ 10 mg/dL as a high inflammatory state, and this threshold has also been presented as a clinically meaningful cutoff in studies of pneumonia severity assessment [23,24]. The stepwise increase in the proportion of high-inflammation episodes (CRP ≥ 10 mg/dL) among mPCR-negative episodes across the pre-pandemic, pandemic, and post-pandemic periods indicates that the increasing inflammatory burden in the negative group was a shift in average level, and was expressed as a growing frequency of clinically meaningful high-inflammatory events. Although the proportion of extreme inflammation (≥P99.5) declined in the post-pandemic period compared with that in the pandemic period, suggesting partial attenuation, the between-period differences remained statistically significant, and the post-pandemic level still exceeded the pre-pandemic baseline. This finding suggests that episodes with extreme inflammatory responses did not fully return to baseline after the pandemic and that some degree of right-tail expansion in the CRP distribution may have persisted. A similar but less pronounced increase was also observed in mPCR-positive episodes. In particular, the increase in CRP-defined inflammatory burden was clearer for CRP ≥ 10 mg/dL than for CRP ≥ P99.5, suggesting that the upward shift across the pandemic transition was not confined to mPCR-negative episodes, although the negative group remained the predominant contributor.

When the virus-specific analyses are considered together with the overall monthly time-series analysis, the epidemiologic tendency toward decoupling between pathogen detection and host inflammatory burden becomes even more apparent. Although detection rates for many viruses declined or remained at low levels during the pandemic and post-pandemic periods relative to the pre-pandemic baseline, the median CRP level increased stepwise over time. These findings suggest that, in the post-pandemic setting, inflammatory burden in clinical practice may not be fully reflected by respiratory virus positivity alone within this hospital-based testing population. However, because detailed data on clinical severity, bacterial co-infection, imaging findings, and comorbid conditions were not comprehensively linked in this dataset, the observed CRP patterns should not be interpreted as direct evidence that one respiratory virus intrinsically caused greater infection severity than another. The virus-specific patterns did not reflect a uniform decline, but rather a heterogeneous restructuring. Whereas HRV, ADV, RSV A/B, influenza B, several coronaviruses, and PIVs predominantly showed decreases or persistence at low levels, some viruses, such as influenza A and PIV-3, showed rebound patterns in the post-pandemic period [25,26]. In our Republic of Korea tertiary-care hospital dataset, this selective rebound occurred in the context of persistently reduced overall positivity and progressively increasing CRP, further supporting that pathogen detection patterns and inflammatory burden followed divergent temporal trajectories rather than returning uniformly to the pre-pandemic state. This suggests that, rather than reflecting simple increases or decreases in individual viruses alone, the post-pandemic period may have involved heterogeneous changes in virus circulation together with increasing inflammatory burden within the clinically tested population. The repeatedly observed negative correlation between monthly positivity rate and median CRP further supports this interpretation. However, this negative correlation should not be generally interpreted as lower viral detection directly implies more severe illness. Rather, it was more likely strengthened by the combination of selection bias and case-mix restructuring during the pandemic and post-pandemic periods, in which the proportion of patients with mild illness decreased while testing became increasingly concentrated among patients with greater severity and higher inflammatory burden. In addition, changes in the burden of underlying comorbidities or distribution of clinical severity among patients presenting after the pandemic, as well as changes in the frequency of secondary bacterial infection or mixed infection, may also have contributed to the increase in median CRP. Therefore, the observed correlation signal is more appropriately interpreted as a descriptive pattern reflecting the combined impact of changes in clinical environment, testing strategy, and patient composition across the pandemic transition, rather than as the consequence of any single causal mechanism.

These findings may have clinical interpretive implications. In the post-pandemic setting, interpreting an mPCR-negative result as an immediate indicator of low risk may have limited explanatory value. Rather, the combination of mPCR negativity and elevated CRP may indicate a subgroup requiring further evaluation, including the possibility of viruses not covered by the panel, bacterial infection, mixed infection, or noninfectious inflammatory conditions. In these patients, a diagnostic pathway that considers microbiological culture, imaging studies, additional molecular testing, and clinical follow-up in parallel may be warranted [27,28]. From the perspective of antimicrobial stewardship, these findings also support cautious interpretation of mPCR positivity or negativity in conjunction with host-response markers, such as CRP, white blood cell count, and procalcitonin, particularly when additional clinical evaluation is required [29,30]. For example, in patients with negative mPCR results but elevated CRP, early integration of culture, imaging, and additional nucleic acid amplification testing may help reduce diagnostic blind spots. Conversely, even in patients with positive mPCR results, when substantial discordance exists between CRP and the clinical presentation, a framework that also considers concurrent bacterial infection or noninfectious inflammatory states may be necessary.

From a surveillance perspective, this study also provides a rationale for moving beyond simple alert systems based solely on detection rates. In the post-pandemic period, adaptive surveillance may be required, with flexible adjustment of testing frequency, target populations, and resource allocation in response to shifting epidemic windows and pathogen redistribution. Therefore, incorporating host-response markers, such as CRP, alongside pathogen detection rates may provide additional contextual information for interpreting post-pandemic surveillance signals [31].

The strengths of this study lie in its long observation window spanning the pre-pandemic, pandemic, and post-pandemic periods; episode-level linkage of mPCR and CRP data; and multilayered analytic design combining monthly time-series analysis with period-specific distributional comparisons. Rather than relying on single-point comparisons, we quantified structural changes at key transition points and their persistence over time, and further characterized the form of decoupling by separately demonstrating the increased burden of high inflammation centered in the mPCR-negative group. By normalizing and comparing pathogen-detection and host-response indicators on the same temporal axis, this study provides structural evidence that the relationship between the two may be reconfigured in a time-dependent manner in the post-pandemic setting.

This study has some limitations. First, because this was a single-center study conducted at a tertiary-care hospital, the generalizability of the findings is limited. In addition, because the analytic dataset was restricted to mPCR episodes with available linked CRP results, selection bias cannot be excluded. Of 23,284 respiratory mPCR episodes identified during the study period, 4282 episodes (18.4%) were excluded because no corresponding CRP result was available. As CRP is more likely to be measured in patients with greater clinical severity or complexity, this inclusion criterion may have enriched the analytic dataset for more severely ill or diagnostically complex cases. Therefore, the findings should be interpreted as reflecting a selected hospital-based population rather than all patients undergoing respiratory mPCR testing during the study period. Second, in this retrospective dataset, detailed patient-level clinical information—including disease severity, symptom onset, hospitalization phase at the time of testing, pre-sampling medication exposure (including antiviral agents, corticosteroids, and antibiotics), microbiological culture results, imaging findings, and comorbidities—was not sufficiently linked, limiting causal decomposition of the etiologies underlying high-inflammatory events and warranting caution in directly interpreting CRP differences as evidence of virus-specific infection severity. Because the principal temporal comparisons and correlation analyses were performed using monthly aggregated data, the observed associations should be interpreted at the aggregate level and not assumed to directly represent patient-level relationships. Third, because referral pathways and the composition of the tested population likely changed before and after the pandemic, it is difficult to completely separate selection bias and case-mix restructuring from true disease-level changes. As the respiratory mPCR platform and panel coverage evolved over the study period, long-term virus-specific detection patterns should be interpreted in the context of these methodological changes as well as underlying epidemiologic shifts, even though internal quality control and standardized laboratory procedures were consistently applied throughout the study period. Fourth, because SARS-CoV-2 was not included in the mPCR panel early in the pandemic and testing pathways also changed during that period, undiagnosed SARS-CoV-2 infection may have been mixed into the mPCR-negative group, which may partly account for the elevated CRP levels observed among negative episodes during the pandemic period. As SARS-CoV-2 test results were not consistently linkable to the study dataset across the entire observation period, we could not quantify the exact contribution of COVID-19 to the inflammatory burden observed in the mPCR-negative group. Additionally, because pathogens not included in the mPCR panel used in this study, including non-panel respiratory viruses and emerging or variant viruses, may have been present, mPCR negativity should not be interpreted as the absence of infection but as a category that may include infections not captured by the diagnostic system in use at the time. Fifth, CRP is a nonspecific inflammatory marker and cannot independently distinguish viral infection, bacterial infection, mixed infection, and noninfectious inflammation. In this study, microbiological data to directly confirm bacterial infection or secondary bacterial infection were not comprehensively linked; therefore, the contribution of these factors could not be quantified. Sixth, as the primary analysis was episode-based, some patients may have contributed more than one testing episode during the study period. However, additional GEE analyses accounting for within-patient clustering, as well as a first-episode sensitivity analysis, showed similar overall directional patterns. These findings suggest that the principal results were not solely driven by repeated observations from the same individual, although some influence on episode-level effect size estimates cannot be entirely excluded. Accordingly, the decoupling observed in this study should be interpreted as a sign of structural change in the pathogen–inflammation relationship, and interpretations attributing it directly to altered pathogen virulence or to any specific single cause should be avoided.

Future studies should focus on determining whether this sign represents a transient phenomenon occurring after an immunity gap or a more durable feature of a changed diagnostic environment. To this end, more detailed clinical timeline data, including symptom onset, hospitalization phase, and treatment exposure before sample collection, should be incorporated to better clarify how diagnostic timing and prior medication use may influence the relationship between respiratory pathogen detection and host inflammatory response. Longer post-pandemic follow-up in multicenter cohorts will be necessary to determine whether the strength of decoupling attenuates, persists, or re-intensifies over time. Additionally, care pathway-based analyses linking primary, secondary, and tertiary care settings may aid in quantifying selection bias and case-mix shifts more directly. Follow-up studies are also needed to directly dissect the etiologic composition of the mPCR-negative/high-inflammation group through integrated models linking mPCR with myxovirus resistance protein A, microbiological culture, imaging, antibiotic prescribing, intensive care unit admission, length of stay, and mortality, as well as through extended molecular diagnostics or metagenomics-based validation incorporating pathogens outside conventional panels and bacterial coinfection.

## 5. Conclusions

Through a 16-year longitudinal time-series analysis encompassing the COVID-19 pandemic, this study provides quantitative evidence that changes in pathogen detection rates based on respiratory mPCR and changes in host inflammatory burden represented by CRP did not fully move in parallel over time. The findings suggest that hospital-based inflammatory burden may not be adequately captured by viral positivity alone across major epidemiologic transitions. These results should be interpreted descriptively and do not establish the causes of CRP elevation. Nevertheless, they indicate that pathogen detection patterns and inflammatory burden may provide complementary information when interpreting respiratory infection trends in real-world clinical practice.

## Figures and Tables

**Table 1:** Respiratory mPCR positivity multiplicity and CRP distribution by pandemic period.

Period	Total Tests, *n*	0 Positive, *n* (%)	1 Positive, *n* (%)	2 Positive, *n* (%)	3 Positive, *n* (%)	4 Positive, *n* (%)	6 Positive, *n* (%)	CRP, Median (IQR), mg/dL
Pre-pandemic (2008–2019)	15,569	6131 (39.4)	7226 (46.4)	1884 (12.1)	300 (1.9)	28 (0.2)	0 (0.0)	0.94 (0.27–3.35)
Pandemic (2020–2022)	2165	1679 (77.5)	342 (15.8)	116 (5.4)	24 (1.1)	4 (0.2)	0 (0.0)	3.35 (0.55–9.44)
Post-pandemic (2023–2024)	1268	939 (74.0)	248 (19.6)	63 (5.0)	14 (1.1)	3 (0.2)	1 (0.1)	5.97 (1.88–13.77)

Percentages are calculated using the total number of tests in each period as the denominator.

**Table 2:** Level changes in monthly testing volume, respiratory virus positivity, and CRP median at the pandemic transition points.

Outcome	Level Change at 2020-01 (95% CI)	*p*-Value	Level Change at 2023-01 (95% CI)	*p*-Value	Unit
Monthly test volume	−51.34 (−70.11 to −32.57)	<0.001	−5.96 (−24.21 to 12.29)	0.5204	episodes/month
Monthly positivity rate	−31.86 (−42.89 to −20.83)	<0.001	5.48 (−3.84 to 14.80)	0.2472	percentage points
Monthly CRP median	2.61 (1.99 to 3.24)	<0.001	3.51 (2.04 to 4.97)	<0.001	mg/dL

**Table 3:** Period-specific proportions of CRP ≥ 10 mg/dL and CRP ≥ P99.5 in multiplex respiratory virus PCR-negative and PCR-positive episodes (2008–2024).

PCR Group	Period	Total (*n*)	CRP ≥10 mg/dL, *n* (%)	CRP ≥ P99.5, *n* (%)
PCR-negative	Pre-pandemic (2008–2019)	6131	845 (13.78%)	17 (0.28%)
PCR-negative	Pandemic (2020–2022)	1679	469 (27.93%)	13 (0.77%)
PCR-negative	Post-pandemic (2023–2024)	939	366 (38.98%)	14 (1.49%)
PCR-positive	Pre-pandemic (2008–2019)	9438	435 (4.61%)	15 (0.16%)
PCR-positive	Pandemic (2020–2022)	486	35 (7.20%)	2 (0.41%)
PCR-positive	Post-pandemic (2023–2024)	329	91 (27.66%)	2 (0.61%)

CRP, C-reactive protein.

## Data Availability

The dataset analyzed in this study was derived from laboratory records at Dankook University Hospital and is subject to institutional as well as national ethical guidelines. Due to confidentiality requirements and data protection regulations, the original raw data cannot be made publicly available. However, de-identified aggregated data may be provided by the corresponding author upon reasonable request and contingent on approval from the Institutional Review Board. All data access inquiries should be directed to the corresponding author.

## Supplementary Materials

The following supporting information can be downloaded at: https://www.mdpi.com/article/10.3390/microorganisms14040908/s1, Table S1: Distribution of respiratory virus detections within the 1-positive and ≥2-positive groups; Table S2: Sensitivity analysis using generalized estimating equations (GEE) for CRP-defined high-inflammatory burden according to pandemic period, accounting for within-patient clustering; Table S3: Sensitivity analysis restricted to the first eligible testing episode per patient; Table S4: Monthly time-series data of multiplex respiratory virus PCR testing volume, positivity, and median CRP concentrations (October 2008–December 2024).

## Abbreviations

The following abbreviations are used in this manuscript:

ADV	Adenovirus
BOCA	Human bocavirus
CRP	C-reactive protein
ETV	Enterovirus
hMPV	Human metapneumovirus
HRV	Human rhinovirus
LIS	Laboratory information system
mPCR	Multiplex PCR
NPI	Non-pharmaceutical interventions
PIV	Parainfluenza
RSV	Respiratory syncytial virus
RV	Respiratory virus
