# Supplementary material for: Decoupling of Respiratory Virus Positivity and Host Inflammatory Response: A 16-Year Longitudinal Study
Source: Microorganisms. 2026 Apr 17;14(4):908. doi: 10.3390/microorganisms14040908 (PMC13118910; doi:10.3390/microorganisms14040908)
Supplement: Supplementary file 1 [file microorganisms-14-00908-s001.zip › microorganisms-4239252-supplementary.pdf]

Table S1 Distribution of respiratory virus detections within the 1-positive and ≥2-positive groups

Group	Virus	n	%
1-positive	HRV	2031	25.99
	RSV A	1097	14.04
	RSV B	887	11.35
	ADV	800	10.24
	Influenza A	762	9.75
	hMPV	541	6.92
	PIV-3	528	6.76
	PIV-1	245	3.13
	Influenza B	233	2.98
	HCoV-OC43	208	2.66
	HCoV-229E	157	2.01
	BOCA	124	1.59
	PIV-2	72	0.92
	ETV	69	0.88
	HCoV-NL63	62	0.79
≥2-positive	HRV	1528	62.70
	ADV	1034	42.43
	PIV-3	376	15.43
	RSV B	371	15.22
	RSV A	363	14.90
	BOCA	278	11.41
	hMPV	261	10.71
	HCoV-OC43	229	9.40
	ETV	210	8.62
	Influenza A	200	8.21
	PIV-1	138	5.66
	HCoV-229E	112	4.60
	Influenza B	64	2.63
	HCoV-NL63	64	2.63
	PIV-2	58	2.38

Table S2. Sensitivity analysis using generalized estimating equations (GEE) for CRP-defined high-inflammatory burden according to pandemic period, accounting for within-patient clustering.

PCR group	Outcome	Comparison	OR (95% CI)	p-value
PCR-negative	CRP ≥10 mg/dL	Pandemic vs pre-pandemic	2.43 (2.13–2.76)	<0.001
PCR-negative	CRP ≥10 mg/dL	Post-pandemic vs pre-pandemic	4.01 (3.45–4.66)	<0.001
PCR-positive	CRP ≥10 mg/dL	Pandemic vs pre-pandemic	1.61 (1.13–2.30)	0.0089
PCR-positive	CRP ≥10 mg/dL	Post-pandemic vs pre-pandemic	7.30 (5.59–9.53)	<0.001
PCR-negative	CRP ≥P99.5	Pandemic vs pre-pandemic	2.53 (1.20–5.32)	0.0148
PCR-negative	CRP ≥P99.5	Post-pandemic vs pre-pandemic	5.53 (2.72–11.30)	<0.001
PCR-positive	CRP ≥P99.5	Pandemic vs pre-pandemic	2.60 (0.59–11.40)	0.2060
PCR-positive	CRP ≥P99.5	Post-pandemic vs pre-pandemic	3.84 (0.88–16.90)	0.0746

Abbreviations: OR, odds ratio; CI, confidence interval; CRP, C-reactive protein; GEE, generalized estimating equation.

Table S3. Sensitivity analysis restricted to the first eligible testing episode per patient.

PCR group	Period	Total (n)	CRP ≥10 mg/dL, n (%)	CRP ≥P99.5, n (%)
PCR-negative	Pre-pandemic (2008–2019)	5251	761 (14.49%)	16 (0.30%)
PCR-negative	Pandemic (2020–2022)	1417	397 (28.02%)	8 (0.56%)
PCR-negative	Post-pandemic (2023–2024)	739	293 (39.65%)	11 (1.49%)
PCR-positive	Pre-pandemic (2008–2019)	7630	366 (4.80%)	12 (0.16%)
PCR-positive	Pandemic (2020–2022)	381	30 (7.87%)	1 (0.26%)
PCR-positive	Post-pandemic (2023–2024)	261	62 (23.75%)	2 (0.77%)

Table S4 Monthly time-series data of multiplex respiratory virus PCR testing volume, positivity, and median CRP concentrations (October 2008–December 2024)

Year	Month	Tests (n)	RV positive (n)	RV positivity rate (%)	CRP median (mg/dL)
2008	10	80	53	66.2	0.58
2008	11	153	117	76.5	0.76
2008	12	160	124	77.5	0.6
2009	1	111	72	64.9	0.85
2009	2	74	39	52.7	1.02
2009	3	99	56	56.6	1.29
2009	4	111	72	64.9	1.27
2009	5	119	79	66.4	1.09
2009	6	86	44	51.2	1.34
2009	7	35	19	54.3	1.81
2009	8	57	21	36.8	2.93
2009	9	77	35	45.5	1.52
2009	10	113	66	58.4	1.14
2009	11	121	81	66.9	1.39
2009	12	129	91	70.5	0.88
2010	1	111	77	69.4	0.53
2010	2	62	34	54.8	0.535
2010	3	88	58	65.9	0.685
2010	4	165	94	57.0	0.87
2010	5	193	121	62.7	0.96
2010	6	136	83	61.0	0.888
2010	7	111	60	54.1	0.77
2010	8	89	69	77.5	1.77
2010	9	82	74	90.2	1.4
2010	10	110	97	88.2	1.12
2010	11	190	147	77.4	0.87
2010	12	251	178	70.9	1.36
2011	1	216	113	52.3	1.7
2011	2	69	39	56.5	1.17
2011	3	102	71	69.6	1.04
2011	4	111	80	72.1	0.96
2011	5	210	153	72.9	0.935
2011	6	107	65	60.7	0.71
2011	7	72	36	50.0	0.766
2011	8	88	21	23.9	1.01

2011	9	119	59	49.6	1.15
2011	10	138	88	63.8	0.505
2011	11	184	103	56.0	0.683
2011	12	110	68	61.8	1.1
2012	1	99	59	59.6	0.74
2012	2	143	94	65.7	1.91
2012	3	136	80	58.8	1.13
2012	4	171	114	66.7	1.44
2012	5	140	94	67.1	1.43
2012	6	106	69	65.1	0.81
2012	7	53	39	73.6	1.16
2012	8	74	38	51.4	0.825
2012	9	62	30	48.4	0.765
2012	10	101	55	54.5	0.59
2012	11	109	77	70.6	0.63
2012	12	106	72	67.9	0.785
2013	1	85	39	45.9	1.95
2013	2	113	71	62.8	1.65
2013	3	182	121	66.5	2.27
2013	4	162	95	58.6	1.39
2013	5	164	76	46.3	1.69
2013	6	134	61	45.5	1.67
2013	7	115	57	49.6	1.21
2013	8	115	49	42.6	1.65
2013	9	103	59	57.3	0.89
2013	10	125	70	56.0	0.97
2013	11	104	56	53.8	0.965
2013	12	105	56	53.3	0.89
2014	1	79	41	51.9	1.12
2014	2	126	85	67.5	1.46
2014	3	127	73	57.5	0.85
2014	4	170	107	62.9	0.99
2014	5	140	88	62.9	0.775
2014	6	92	35	38.0	1.35
2014	7	76	40	52.6	0.485
2014	8	106	53	50.0	0.445
2014	9	100	50	50.0	0.54
2014	10	73	35	47.9	1
2014	11	119	85	71.4	0.76

2014	12	184	125	67.9	0.6
2015	1	85	51	60.0	0.48
2015	2	91	65	71.4	0.8
2015	3	111	65	58.6	0.73
2015	4	134	103	76.9	0.515
2015	5	138	88	63.8	0.815
2015	6	56	29	51.8	0.625
2015	7	73	34	46.6	0.91
2015	8	90	36	40.0	1.26
2015	9	81	37	45.7	0.7
2015	10	105	58	55.2	1
2015	11	116	73	62.9	0.845
2015	12	192	155	80.7	0.8
2016	1	99	63	63.6	0.72
2016	2	113	70	61.9	1.27
2016	3	108	83	76.9	0.61
2016	4	142	95	66.9	1.16
2016	5	143	108	75.5	0.94
2016	6	106	68	64.2	0.815
2016	7	139	96	69.1	0.88
2016	8	91	48	52.7	1.17
2016	9	70	37	52.9	0.92
2016	10	106	70	66.0	0.88
2016	11	183	148	80.9	0.44
2016	12	192	145	75.5	0.62
2017	1	86	50	58.1	0.705
2017	2	77	45	58.4	0.75
2017	3	110	75	68.2	0.625
2017	4	106	81	76.4	0.725
2017	5	100	75	75.0	0.51
2017	6	76	41	53.9	0.97
2017	7	79	43	54.4	0.48
2017	8	61	33	54.1	0.86
2017	9	77	51	66.2	0.46
2017	10	104	66	63.5	0.625
2017	11	155	106	68.4	0.46
2017	12	162	129	79.6	0.62
2018	1	138	90	65.2	0.56
2018	2	84	47	56.0	1.1

2018	3	98	57	58.2	0.625
2018	4	97	57	58.8	0.8
2018	5	120	90	75.0	0.53
2018	6	113	59	52.2	0.9
2018	7	116	74	63.8	0.56
2018	8	99	44	44.4	1.11
2018	9	87	51	58.6	0.64
2018	10	80	41	51.2	0.89
2018	11	111	74	66.7	1.16
2018	12	217	156	71.9	0.73
2019	1	153	69	45.1	2.37
2019	2	117	38	32.5	2.02
2019	3	120	40	33.3	1.2
2019	4	120	64	53.3	0.97
2019	5	141	78	55.3	0.79
2019	6	132	51	38.6	1.4
2019	7	110	39	35.5	1.4
2019	8	112	34	30.4	2.05
2019	9	110	41	37.3	1.66
2019	10	97	41	42.3	1.35
2019	11	113	44	38.9	1.81
2019	12	89	34	38.2	2.28
2020	1	134	73	54.5	2.99
2020	2	114	36	31.6	3.39
2020	3	52	4	7.69	3.16
2020	4	62	0	0.0	3.42
2020	5	52	3	5.77	5.88
2020	6	39	1	2.56	4.99
2020	7	59	6	10.2	4
2020	8	45	9	20.0	3.92
2020	9	49	4	8.16	6.84
2020	10	56	2	3.57	5.06
2020	11	70	9	12.9	3.64
2020	12	51	7	13.7	3.58
2021	1	33	2	6.06	5.97
2021	2	47	3	6.38	3.39
2021	3	51	4	7.84	2.59
2021	4	32	4	12.5	3.3
2021	5	47	12	25.5	1.52

2021	6	41	8	19.5	3.36
2021	7	44	4	9.09	3.42
2021	8	42	3	7.14	3.81
2021	9	62	9	14.5	5.71
2021	10	69	31	44.9	1.26
2021	11	79	33	41.8	1.88
2021	12	55	22	40	2.55
2022	1	66	41	62.1	0.47
2022	2	54	29	53.7	0.3
2022	3	52	7	13.5	4.46
2022	4	45	1	2.22	5.2
2022	5	64	9	14.1	3.58
2022	6	56	10	17.9	3.96
2022	7	74	23	31.1	1.68
2022	8	99	24	24.2	4.03
2022	9	84	19	22.6	3.15
2022	10	65	10	15.4	4.71
2022	11	72	10	13.9	4.67
2022	12	49	14	28.6	3.93
2023	1	64	21	32.8	3.87
2023	2	52	7	13.5	4.5
2023	3	87	32	36.8	4.54
2023	4	90	39	43.3	2.94
2023	5	87	31	35.6	4.67
2023	6	97	28	28.9	3.74
2023	7	79	19	24.1	5.81
2023	8	99	19	19.2	6.34
2023	9	44	4	9.09	10.2
2023	10	53	10	18.9	9.41
2023	11	55	10	18.2	11.8
2023	12	71	26	36.6	7.7
2024	1	96	32	33.3	9.44
2024	2	58	14	24.1	10.5
2024	3	19	4	21.1	8.32
2024	4	36	5	13.9	3.68
2024	5	33	7	21.2	8.86
2024	6	32	7	21.9	10.3
2024	7	30	4	13.3	9.38
2024	8	23	3	13.0	10.8

2024	9	13	0	0.0	5.33
2024	10	14	2	14.3	5.31
2024	11	19	0	0.0	5.51
2024	12	17	5	29.4	7.48

Abbreviations: CRP, C-reactive protein; RV, respiratory virus.

tests_n, total number of multiplex respiratory virus PCR tests performed in each month; RV positive (n), number of PCR-positive tests; RV positivity rate (%), monthly positivity rate (%), calculated as (pos_n/tests_n) × 100; crp_median (mg/dL), monthly median CRP concentration (mg/dL). This table provides the monthly aggregated dataset used for descriptive and time-series analyses.
